# Sea cucumber (*Acaudina leucoprocta*) peptides extended the lifespan and enhanced antioxidant capacity via DAF-16/DAF-2/SOD-3/OLD-1/PEPT-1 in *Caenorhabditis elegans*

**DOI:** 10.3389/fnut.2022.1065145

**Published:** 2022-11-22

**Authors:** Yue Wu, Jingjuan Yang, Chengmei Xu, Qiuqi Li, Yage Ma, Shenglan Zhao, Jiachen Zhuang, Fei Shen, Qianqian Wang, Fengqin Feng, Xi Zhang

**Affiliations:** ^1^School of Traditional Chinese Medicine, Yunnan University of Traditional Chinese Medicine, Kunming, China; ^2^School of Biosystems Engineering and Food Science, Zhejiang University, Hangzhou, China

**Keywords:** sea cucumber peptide, *Caenorhabditis elegans*, anti-aging, antioxidant, insulin/IGF-1 signaling

## Abstract

The sea cucumber peptides (SCPs) from *Acaudina leucoprocta* were derived from the patented bio-enzyme digestion technology and the molecular weight of obtained SCPs was < 10 kDa. In this study, we investigated the possible anti-aging effects of SCPs on the model of *Caenorhabditis elegans* and the underlying mechanisms. SCPs extend the average lifespan of nematodes by 31.46%. SCPs enhance the anti-stress capacity of *C. elegans* by improving heat resistance and mobility, Also, the accumulated potential oxidative stress inducers like lipofuscin and reactive oxygen species (ROS) were reduced to 40.84 and 71.43%. In addition, SCPs can increase the antioxidant capacity in nematodes by enhancing the activity of SOD and CAT and reducing MDA accumulation in nematodes to 32.44%. Mechanistically, SCPs could mediate DAF-16/DAF-2/SOD-3/OLD-1/PEPT-1 axis to improve antioxidant capacity and extend lifespan in nematodes. Taken together, these findings provide a direction for the anti-aging effects of sea cucumber peptides and new insights into the further purifications of SCPs and future research on aging.

## Introduction

Aging is typically characterized as the slash of the body's physical and psychological adaptability to outside stress, which is induced by the combined actions of holistic physiologic factors like stem cell decline, DNA degradation, and external dietary and mental factors (1, 2). During the aging process, the functions of multiple organisms will decline, which could induce aging-related diseases such as hypertension, hyperlipidemia, diabetes, neurodegenerative diseases, cardiovascular diseases, and even worse, cancer (3). Therefore, healthy life extension effectively delays aging and reduces the prevalence of aging-related diseases (4).

In ancient times, sea cucumber was a precious food for nutrition supplementation and physical fitness elevation. According to *Compendium of Materia Medica*, sea cucumber is endowed with warm and nourishing properties, and its effects are almost the same as that of ginseng whose nourishing effect on the kidney meridian, has been well-documented, benefits the essence, dispel phlegm and saliva, diuretic, invigorate blood and impotence, and cure the spread of ulcers (5). *Acaudina leucoprocta* is a kind of edible sea cucumber distributed in Zhejiang, Guangdong, Fujian Province, and northwest Australia, which has become a new species of economic interest in China for its untapped nutrient potential and its abundance (6). *Acaudina leucoprocta* is abundant and low-cost, with proven technology for heavy metal removal (7), providing a low-cost alternative to the current expensive peptide resources. Sea cucumber peptides are frequently useful for preparing soup and drinking in our life (8). Sea cucumber peptides (SCPs) obtained from *A. leucoprocta* by protease hydrolysis, and isolated and purified by neutral proteinase complex are small molecule peptides (9). The study of the bioavailability of bioactive peptides after *in vitro* digestion and first metabolism revealed that all peptides were transported across the intestinal cell layer to varying degrees, and are not metabolized by the liver (10). It has also been shown that the diversity of peptides increases after *in vitro* gastric and small intestinal digestion (11). Therefore, the peptides are stable, not metabolized, and not degraded during human digestion, increasing diversity. It has been reported that these kinds of SCPs are endowed with anti-oxidation, anti-aging, anti-fatigue, and immunomodulatory properties (12). Moreover, sea cucumber peptides with a high degree of hydrolysis obtained by biological enzymatic hydrolysis have excellent solubility, stability, emulsification, easy digestion, and absorption characteristics good nourishing product (13).

*C. elegans*, a classical model organism, has a clear genetic background, a body structure accessible for detection, and short life history. It applies to utilizing molecular biology methods for genetic intervention and behavioral tracing (14). In *C. elegans*, IIS plays a key role in the regulation of development, metabolism, and aging (15). The IIS is a key pathway in the delayed aging of SCPs with the following key genes, (*daf-16, daf-2, sod-3*). SODs acted as a superoxide anion radical remover, which could respond to oxidative stress and affect the lifespan of nematodes (16). In addition, *daf-2* is a key gene for ROS resistance and increased lifespan (17). Research shows that *daf-16* is a downstream gene of *daf-2* and an upstream gene of *sod-3* (18, 19). The *old-1* and *pept-1* are closely linked to insulin pathway-related genes. OLD-1 signaling was specific for longevity and stress resistance and may transduce signals to DAF-16 (20, 21). PEPT-1 is an electrogenic symporter that couples substrate transport to proton movement across the membrane, therefore leading to an acidification of the cytosol (22). Genetic studies indicated that *pept-1* interacts with both the *daf-2*/insulin- and *let-363*/TOR-signaling pathways to regulate lifespan and with the *daf-2* pathway to influence stress response (23). Therefore, nematodes were used to study the anti-aging activity of SCPs and their mechanism. However, the possible effects of SCPs on DAF-16/DAF-2/SOD-3/OLD-1/PEPT-1 remain unelucidated.

We examined the effect of SCPs on the lifespan and stress resistance of *C. elegans*. The discovery of the anti-aging effect and the mechanism of sea cucumber peptides (*Acaudina leucoprocta*), proposed a new direction for future research on peptide-related anti-aging mechanisms: DAF-16/DAF-2/SOD-3/OLD-1/PEPT-1, and also provided the basis for the purification of sea cucumber peptides in the future.

## Materials and methods

### Strains and reagents

Sea cucumber peptides were provided by Hangzhou Kang Yuan Food Technology Co Ltd (Hangzhou, CHN), Metformin (Met), β-nicotinamide mononucleotide (NMN), Methyl viologen (paraquat), and 5-fluoro-2'-deoxyuridine (FUDR) and were obtained from Shanghai Aladdin Biochemical Technology Co., Ltd. (Shanghai, CHN). Total superoxide dismutase (SOD) assay kit, catalase (CAT) assay kit and malondialdehyde (MDA) assay kit were provided by Nanjing Jiancheng Bioengineering Institute (Nanjing, CHN). Reactive oxygen species (ROS) was obtained from Vigorous Biotechnology Beijing Co., Ltd. (Beijing, CHN).

*C. elegans* (wild-type N2), and mutant strains CF1038 [*daf-16(mu86)I*], TJ356 [zIs356 IV (*daf-16p::daf-16a/b::GFP* + *rol-6(su1006)*)], CB1370 [*daf-2(e1370)III*], CF1553 [(pAD76) *sod-3p::GFP*], SN5[*old-1(mk1)II*], BR2742[*pept-1(lg601)X*.], RB1159[*tyr-3(ok1194)I*.], RB1985[*acox-1.5(ok2619) III*.].were purchased from the Caenorhabditis Genetics Center (CGC). The expression of Green Fluorescent Protein (GFP) and the lifespan of the mutant nematodes were recorded. Escherichia coli OP50 (E. coli OP50) was obtained from SunyBiotech.

### Determination of amino acid composition

Gel exclusion chromatography in HPLC was used to TSK-GEL G2500PWXL as the separation column to analyze the molecular weight distribution of SCPs. The amino acid composition of SCPs was measured by HPLC (Thermo Scientific, Waltham, USA). Briefly, 20 mg of samples were dissolved in 6 M HCl (1 mL), followed by hydrolysis at 150°C for 1.5 h. After the completion of hydrolysis, derivation. After filtration with a 0.22 μm membrane filter, the sample was analyzed by HPLC on the AQ-C18 column (4.6 × 250 mm, Welch Co., Ltd, Shanghai, China) at 254 nm using the mobile phase of A (acetic acid/sodium acetate buffer solution) and B (80% acetonitrile) at 1.0 mL/min.

### Cultivation of *C. elegans*

The L4 stage of nematodes was transferred to NGM plates containing SCPs-L (0.0625 mg/mL), SCPs-M (0.125 mg/mL), SCPs-H (0.25 mg/mL), Metformin (Met, 40 μmol/L), β-nicotinamide mononucleotide (NMN, 0.25 mg/mL) and FUDR (1 mg/mL) to prevent spawning. For the synchronization of *C. elegans*, put the pregnant adults in the NGM medium coated with OP50 and observe their oviposition status. The pregnant adults are picked out after oviposition, and the eggs are cultured for 48 h to L4 stage adults at 20°C to get the same-aged worms for further experiments (16).

### Lifespan assay

All lifespan assays were carried out at 20°C. The transfer day was designated as day 0. Worms were transferred another day to fresh extracts or control plates until day 7, which was thought to be the adulthood of the *C. elegans*. During the experiment, worm bags and abnormal dead worms were removed. Triplicate plates were used for each group (30 per plate). During the lifespan test, the movements of the *C. elegans* were recorded at 18, 20, 22, and 24 days.

### Stress test

Same-aged *C. elegans* were picked onto the medicated plates and cultivated for 7 days. For the thermotolerance assay, nematodes were placed in 37°C conditions for 13 h and then the number of dead worms was counted. Triplicate plates were used for each group (30 per plate). To test the resistance of *C. elegans* to paraquat, nematodes were exposed to 140 mM paraquat. The number of surviving worms was counted every 2 h until all the worms died.

### Lipofuscin determination

To determine the lipofuscin level, on 13 days of treatment, worms treated with SCPs were placed on 1% agarose pads on glass slides and anesthetized with Levamisole Phosphate (6.75 μM). The worms were visualized under a fluorescence microscope (Axio Scope. A1, American), and were measured using Image J 1.8.0 software.

### ROS detection

After day 7 of treatment with SCPs, the nematodes were washed with nutrient buffers (M9), and then 100 μL of the prepared ROS fluorescent agent was added for fluorescent excitation at 20°C for 5 h. The worms were visualized under a fluorescence microscope (Axio Scope. A1, American). The fluorescence intensity of the worms was measured using Image J 1.8.0 software (24).

### Antioxidant detection in *C. elegans*

The nematodes treated by SCPs were lysed with double distilled water in an ice bath after 7 days. The activities of SOD, MDA, and CAT content were determined (25).

### Real-time quantitative PCR

Total RNAs of nematodes administration (7 days) were isolated using an RNA extraction kit (Aidlab Biotechnologies Co. Ltd, Beijing, China) and cDNAs were synthesized using a reverse transcription kit (Takara, Biotechnology Co. Ltd, Dalian, China). The qPCR reaction was performed on a qTOWER 2.0 PCR system (Light Cycler, Switzerland) using SYBR Green PCR Master Mix (Takara). Relative expression levels of genes were calculated using the 2^−Δ*ΔCT*^ method, and the gene *act-1* was set as the internal reference, shown in Table 1. The best primers for genes of *C. elegans* were obtained from the qPrimerDB-qPCR Primer Database (26).

**Table 1:** Primer sequences.

**Gene**	**Gene ID**	**Direction**	**Primer sequences (5^′^-3^′^)**
*Caenorhabditis elegans act-1*	1,79,535	Forward	GCAAGAATACGACGAGTCCG
		Reverse	TAGAAAGCTGGTGGTGACGA
*Caenorhabditis elegans daf-2*	1,75,410	Forward	CCCAAGTTTGAGCTCCAAGAG
		Reverse	TCGTCATCGTTCTGTCTGCAT
*Caenorhabditis elegans daf-16*	1,72,981	Forward	GCTGCTGCCTTCACTCTCAT
		Reverse	GATGACTGGCCACTGGTGTG
*Caenorhabditis elegans sod-3*	1,81,748	Forward	TCTCCAACCAGCGCTGAAAT
		Reverse	CCAGAGCCTTGAACCGCAAT
*Caenorhabditis elegans old-1*	1,71,737	Forward	CACCAGAAAGCTCCGTTCAGA
		Reverse	ACTGAGGAAGAGGAATCAAGTG
*Caenorhabditis elegans pept-1*	1,80,919	Forward	AAACTTTGCCATGTGCCGTC
		Reverse	ACAGCCGGTTGGGAACTAAG
*Caenorhabditis elegans tyr-3*	1,72,472	Forward	TCATGCGCCCATTTACACCA
		Reverse	CGAGGAGCTCCGTGTGATTT
*Caenorhabditis elegans acox-1.5*	1,76,353	Forward	AACTGAGTGGTGGCTGATGG
		Reverse	GATTGGTTCCGTGTCCGAGT

This table includes all primers used in RT-qPCR experiments.

### Statistical analysis

Data are reported as means ± standard deviations (SD) or standard errors (SEM). Assays for each experiment were performed independently at least three times. At least three separate assays were run for each experiment. The Kaplan-Meier test was used to plot survival curves using GraphPad Prism 8.0 (GraphPad Software, Inc., CA, USA), and SPSS 21.0 was used to do the log-rank test. One-way analysis of variance (ANOVA) analyses with the Tukey-Kramer test or *t*-test was carried out to detect the statistical significance with a significant level of α = 0.05 (27).

## Results

### Amino acids composition and Molecular weight distribution of SCPs

**Figure 1:**
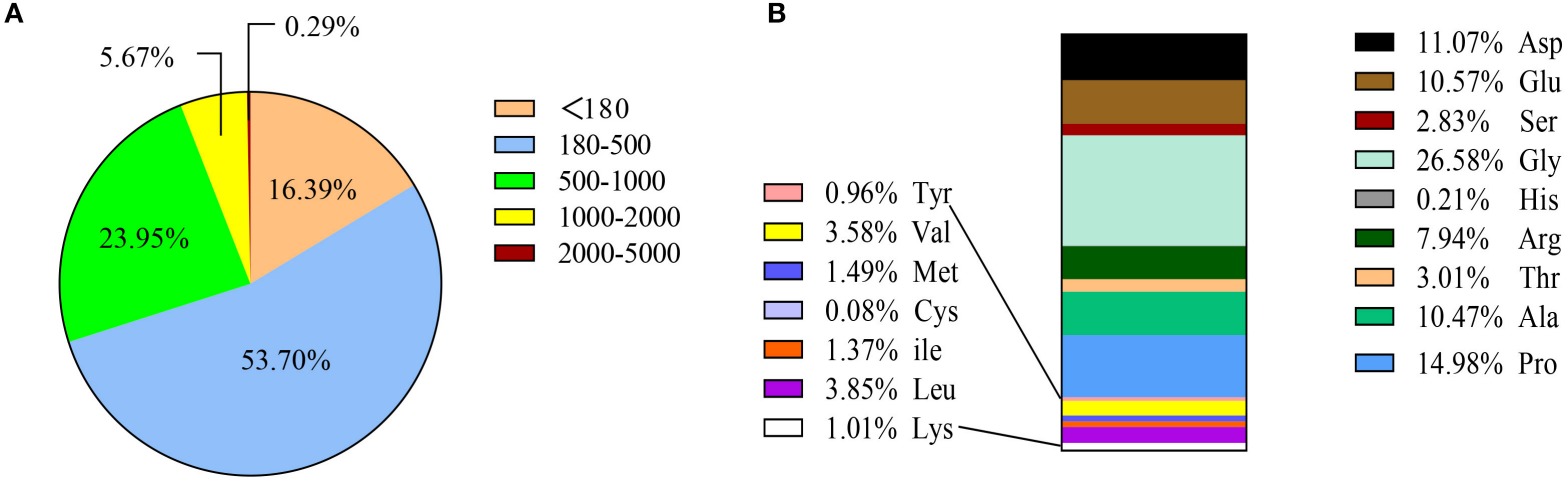
Amino acid composition and MW distribution of SCPs. **(A)** MW distribution of SCPs. **(B)** Amino acid compositions of SCPs.

The molecular weight of SCPs < 1,000 Da accounted for 94.04% of the total, of which molecular weights < 180 Da, 180–500 Da, and 500-1000 Da respectively accounted for 16.39, 53.70, and 23.95%, and molecular weight >2,000 Da only accounted for 0.29%. This shows that sea cucumber peptides are small molecule peptides that are helpful for human digestion and absorption. SCPs are also high in glycine (Gly), proline (Pro), aspartic (Asp), and Alanine (Ala) (Figure 1).

### SCPs extended the lifespan of *C. elegans*

**Figure 2:**
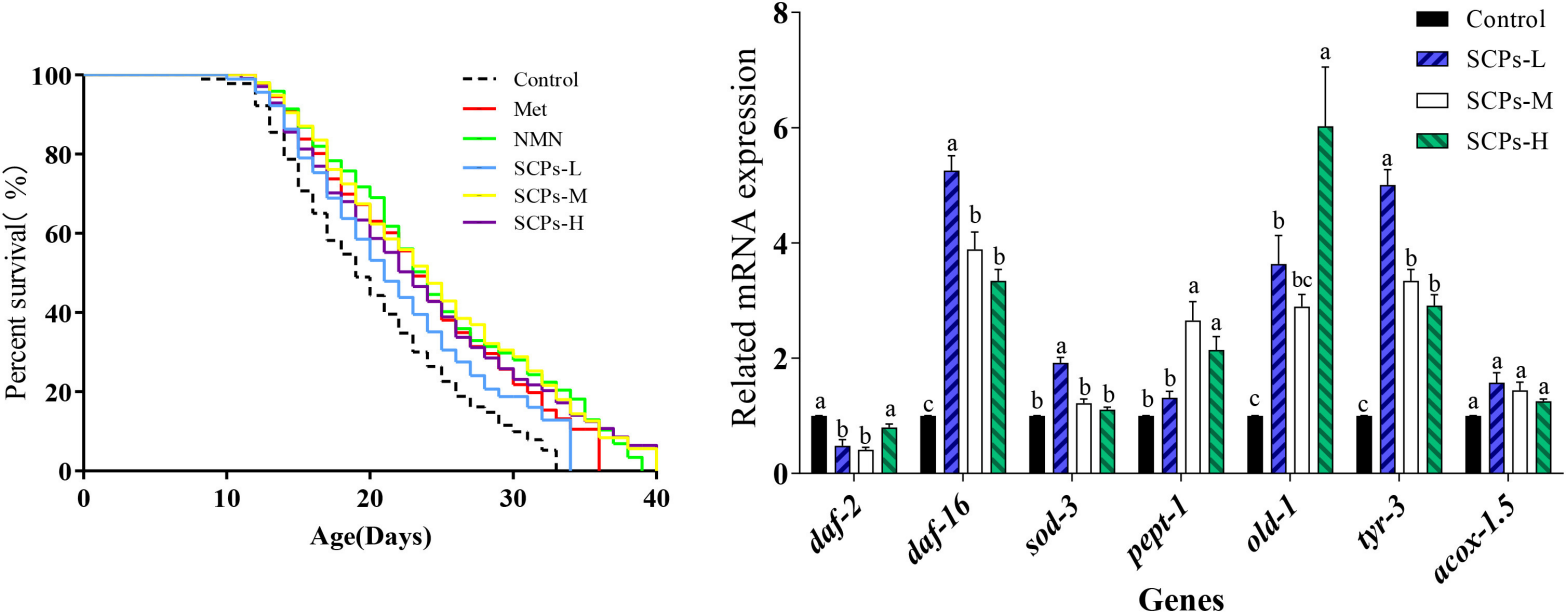
Effects of SCPs on the lifespan of wild-type *C. elegans* under normal conditions. Statistical analyses were carried out using GraphPad software. A Kaplan-Meier lifespan analysis was carried out, and *p-values* were calculated using the log-rank test. In all statistical analyses, *p* < 0.05 was accepted as statistically significant.

The results showed that the survival rate of the nematodes pretreated with SCPs was much higher than the control group (Figure 2). As shown in Table 2, the mean lifespan of SCPs-L, SCPs-M, and SCPs-H groups was increased by 6.50, 31.46, and 21.30%, compared to the control group. Meanwhile, the maximum lifespan of the three groups was increased by 3.48, 32.17, and 20.00%, compared to the control group. More importantly, after administering SCPs-M, the mean and maximum lifespans were prolonged by 21.09 and 2.86%, 21.74, and 10.43%, compared to the Met and NMN groups.

**Table 2:** Effects of SCPs on the life span of wild-type *C. elegans* under normal conditions.

**Strain (solvent)**	**Maximum lifespan (d)**	**Life extension rate (%)**	**Mean lifespan (d)**	**Life extension rate (%)**
N2 (Control)	28.75 ± 1.89^b^	0	17.81 ± 1.39^b^	0
N2 (Met)	31.75 ± 2.63^ab^	10.43	19.65 ± 3.02^ab^	10.37
N2 (NMN)	35.00 ± 5.89^ab^	21.74	22.90 ± 1.46^a^	28.60
N2 (SCPs-L)	29.75 ± 3.40^ab^	3.48	19.56 ± 2.35^ab^	6.50
N2 (SCPs-M)	38.00 ± 4.20^a^	32.17	23.41 ± 1.91^a^	31.46
N2 (SCPs-H)	34.50 ± 4.35^ab^	20.00	21.60 ± 2.60^ab^	21.30

Different letters correspond to statistically significant differences (*p* < 0.05) between groups in the same column.

### SCPs enhanced the stress tolerance and motion ability of *C. elegans*

**Figure 3:**
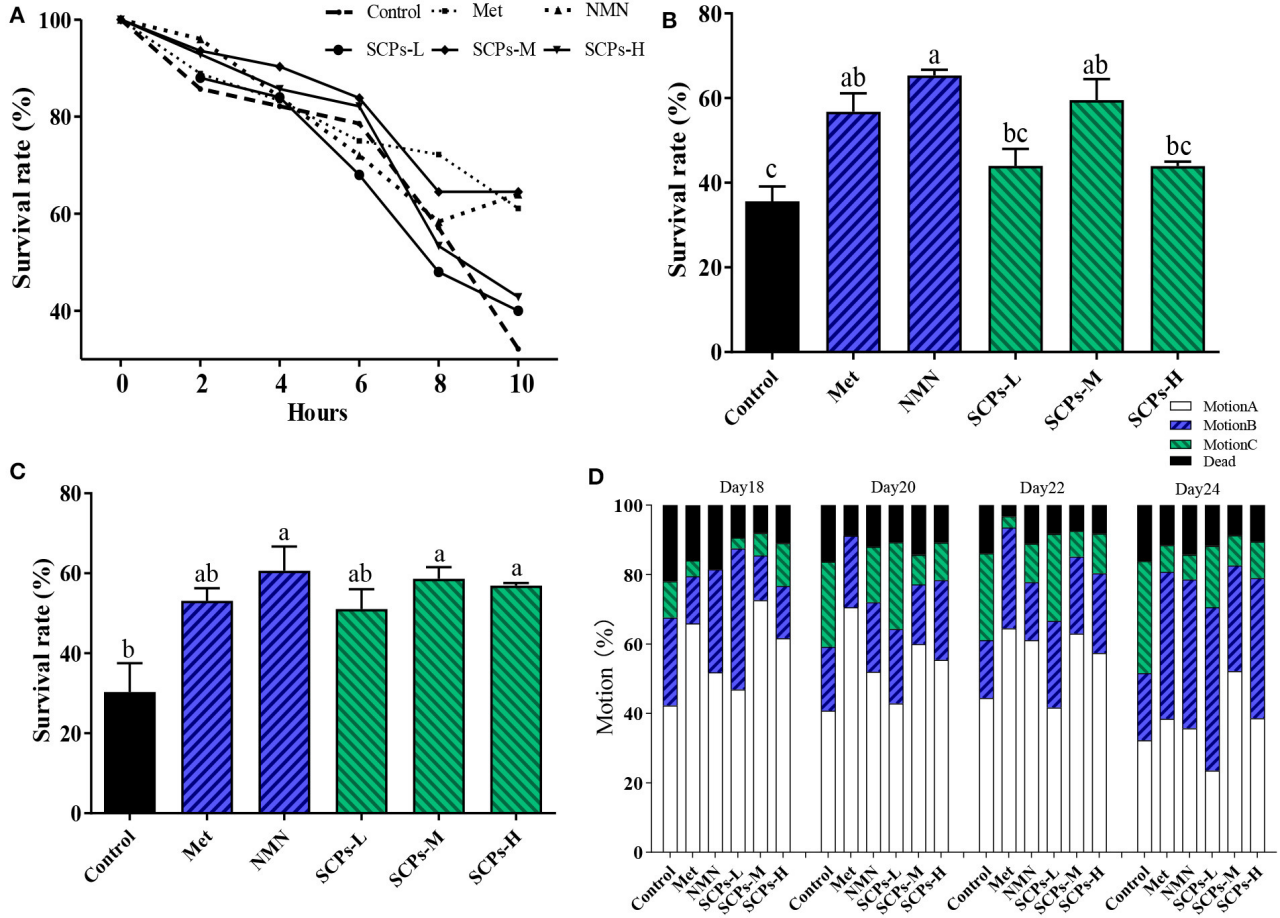
Effects of SCPs on the stress and motion ability in N2 over time. **(A)** Effect of SCPs on N2 survival over time under paraquat-stimulated. **(B)** Effect of SCPs on N2 survival under paraquat-stimulated. **(C)** Effect of SCPs on the survival of N2 at 37 °C thermal shocks. **(D)** The effect of SCPs on the mobility of N2. The movement is the free crawling as action A; the body twisting or head twisting after the platinum wire is lightly touched as action C; between the two is action B. Different letters correspond to statistically significant differences (*p* < 0.05) between groups.

At 10 h, there was a significant difference between the levels of SCPs-M and positive controls were consistent (Figure 3A). The survival rates of SCPs groups were considerably greater than the control group during paraquat stress and heat stress (Figures 3B,C). At the same time, we detected whether the increased lifespan was accompanied by an improvement in the vitality of the nematodes. We tested the motility of nematodes treated with SCPs for 18, 20, 22, and 24 days, as shown in Figure 3D. The mortality of the SCPs treatment group was lower than the control group, which suggested that SCPs did not affect muscle contraction and other functions of N2. The majority of worms could move on their own by day 18. By day 20, only 40.81% of the worms in the control group possessed class A motility, compared to 42.86, 60.00, and 55.41% in the SCPs-L, M, and H treatment groups, respectively. On days 22 and 24, 24.07 and 30.99% of worms had class A and B motility in the SCPs-M treatment group more than those in the control group. These findings suggested that SCPs could improve *C. elegans* capacity for mobility and stress tolerance.

### SCPs increased the antioxidant effect and decreased lipofuscin accumulation of *C. elegans*

**Figure 4:**
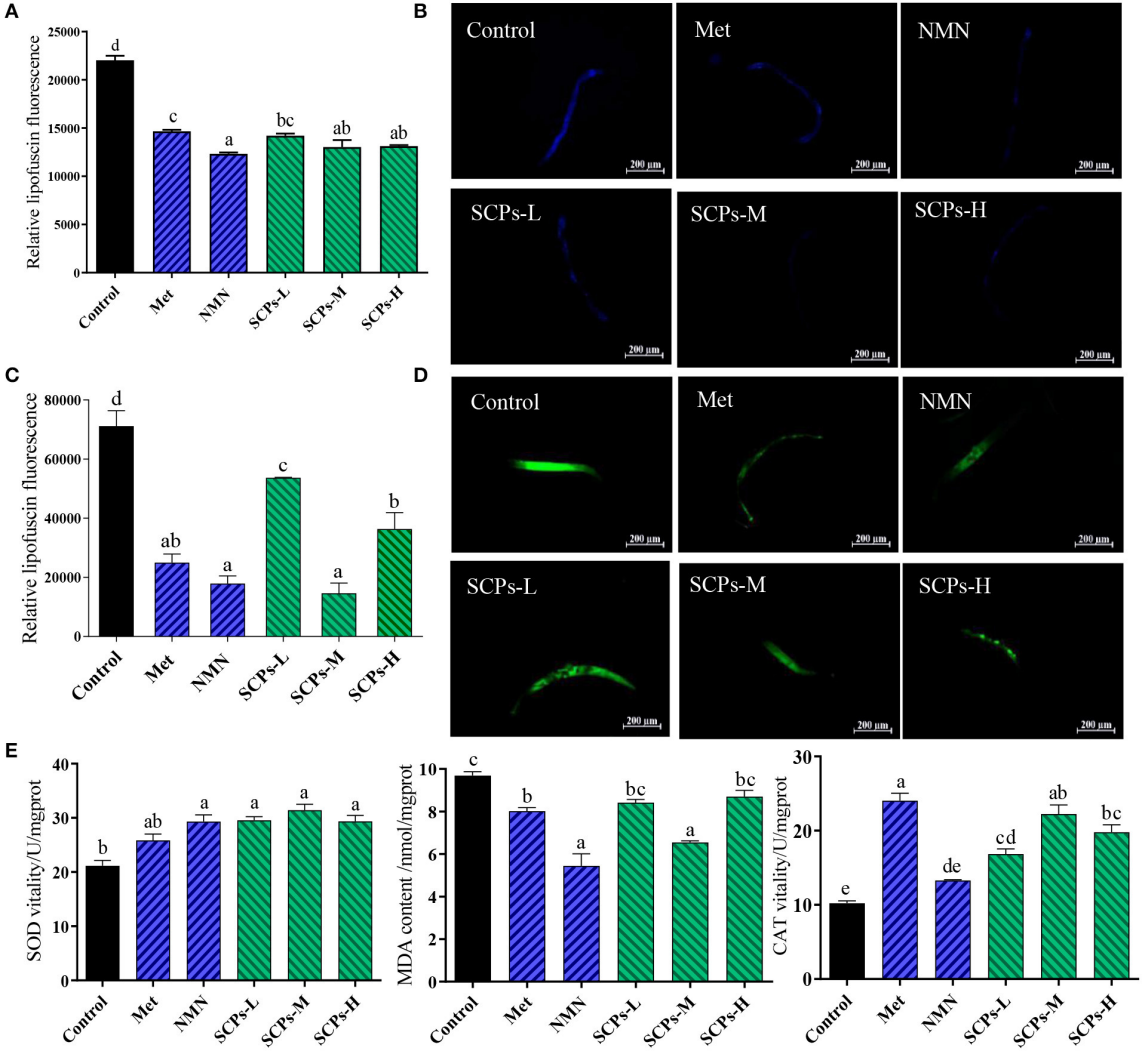
Effect of SCPs on lipofuscin, ROS accumulation, and internal oxidation in N2. **(A)** The bar shows a gray value on lipofuscin in N2 after treatment with SCPs. **(B)** The typical fluorescent pictures of lipofuscin. **(C)** The bar shows a gray value on ROS in N2 after treatment with SCPs. **(D)** The typical fluorescent pictures of ROS accumulation. **(E)** The effects of SCPs on SOD, MDA, and CAT of N2 under normal conditions. Different letters correspond to statistically significant differences (*p* < 0.05) between groups.

The autofluorescence of lipofuscin can be used to estimate the aging of nematodes. As shown in Figures 4A,B, SCPs, Met, and NMN can effectively reduce lipofuscin in nematodes. The lipofuscin content in N2 worms treated with SCPs-L, M, and H was lower by 35.48, 40.84, and 40.41%, respectively, compared to the control group. Increased ROS levels may damage cell structure significantly, so prevention of excessive ROS accumulation has been shown to be an efficient strategy to delay aging. When the nematodes were pretreated with SCPs, the fluorescence of N2 was substantially reduced as compared to the control group. Among these, the SCPs-M group had the lowest ROS content at the same level as the NMN group (Figures 4C,D). The activities of SOD, MDA, and the CAT contents are important indexes of defense against potential oxidative damage in organisms. As shown in Figure 3E, SOD activities were significantly increased by SCPs treatment (by 1.4–1.5-fold in the SCPs-treated groups compared with the control group). The MDA contents were 10.18%-32.44% lower in the SCPs treated groups than in the control group. The CAT activities were significantly increased by SCPs treatment (by 1.6–2.1-fold in the SCP-treated groups compared with the control). These results indicated that SCPs reduced oxidative deposition in the body and enhanced the antioxidant capacity of nematodes.

### SCPs up-regulated *daf-16, sod-3, pept-1, old-1, tyr-3, acox-1.5*; down-regulated *daf-2*

**Figure 5:**
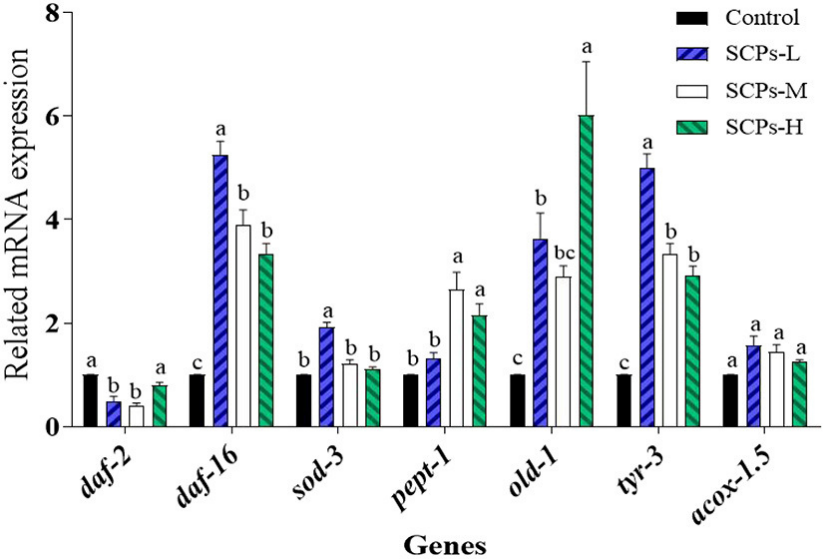
Effect of SCPs on the relative expression levels of anti-aging related genes in *C. elegans*. Data were expressed as the mean ± SD. Different letters correspond to statistically significant differences (*p* < 0.05) between groups in the same gene.

To explore the mechanisms of these beneficial effects, we analyzed the expression levels of genes related to longevity, stress resistance, apoptosis, or the impact Dauer period (Figure 5). The expression level of genes in the control group was set to 1. In SCP s-treated groups, the relative expression levels of *daf-2* ranged from 0.41 ± 0.08 to 0.79 ± 0.11, *daf-16* and *sod-3* were SCPs-dose-dependent; the relative expression levels of *pept-1* ranged from 1.31 ± 0.20 to 2.65 ± 0.57. Those of *old-1* ranged from 2.89 ± 0.37 to 6.02 ± 1.78. Other genes showing increased expression levels after SCPs treatment included *tyr-3*, and *acox-1.5*. It is hypothesized that *pept-1* may regulate *daf-16* and its down-regulated genes through *daf-2* (28).

### *Daf-16, daf-2, sod-3, pept-1*, and *old-1* genes were required for the benefits of SCPs in *C. elegans*; *tyr-3* and *acox-1.5* genes were not required for the benefits of SCPs in *C. elegans*

**Figure 6:**
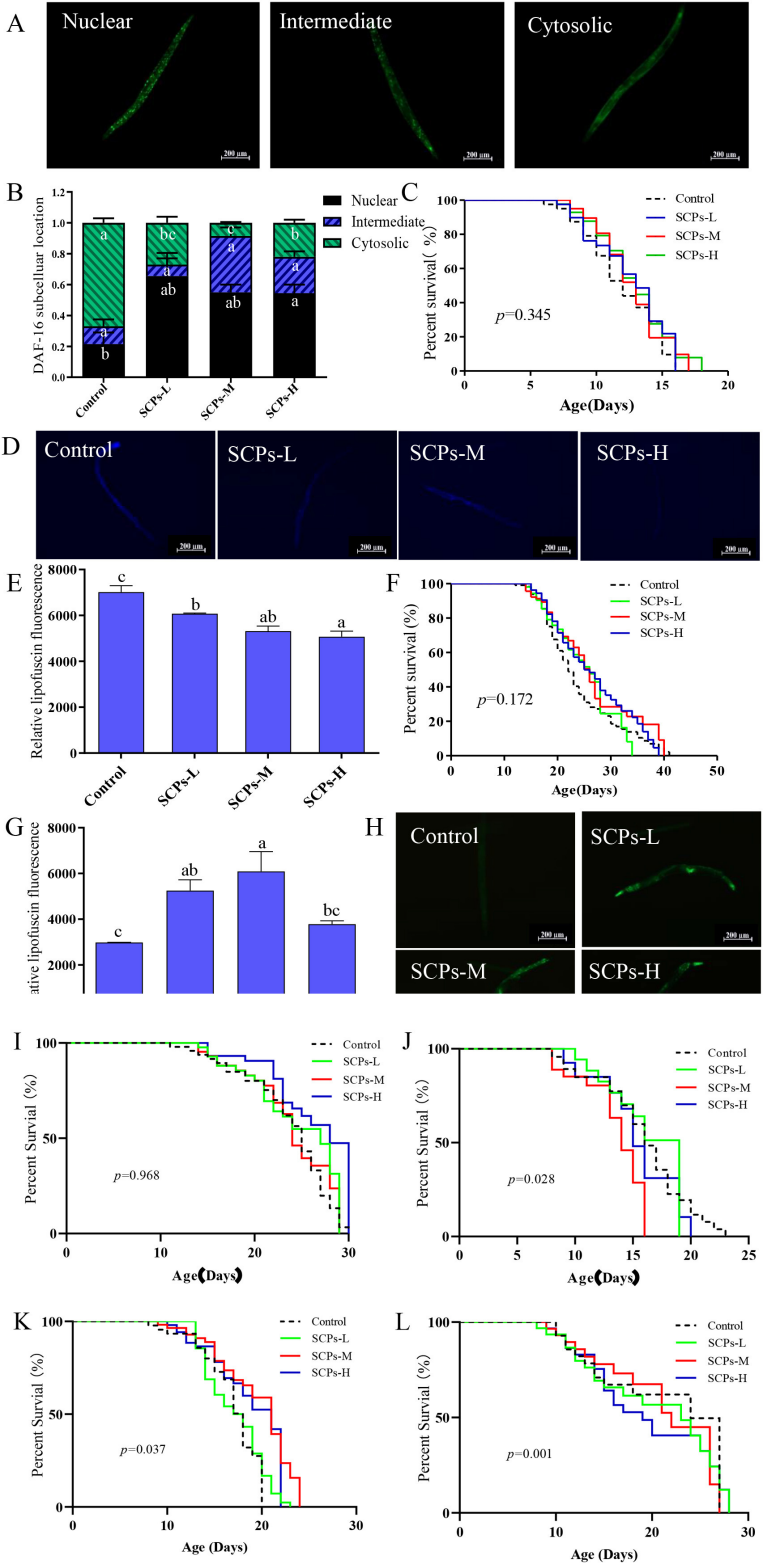
SCPs extended the lifespan of *C. elegans* through the *daf-2,daf-16*,*sod-3*,*old-1*,*pept-1*. SCPs extended the lifespan of *C. elegans* and could not through the *tyr-3* and *acox-1.5*. **(A)** Distribution of DAF-16 “cytosolic,” “intermediate” and “nuclear,” **(B)** The proportion of DAF-16: GFP “cytosolic,” “intermediate” and “nuclear,” **(C)** Effect of SCPs on the lifespan of *daf-16* mutants. **(D)** The lipofuscin accumulation was presented in fluorescent pictures. **(E)** The bar shows a gray value on lipofuscin in *daf-2(e1370)* after treatment with SCPs. **(F)** Effect of SCPs on the lifespan of *daf-2(e1370)* mutants. **(G)** The bar shows a gray value on fluorescent in (pAD76) *sod-3p: GFP* after treatment with SCPs. **(H)** Image of the fluorescence intensity in CF1553. **(I)** Effect of SCPs on the lifespan of *pept-1(lg601) X*. mutants. **(J)** Effect of SCPs on the lifespan of *old-1(mk1) II* mutants. **(K)** Effect of SCPs on the lifespan of RB1159[*tyr-3(ok1194) I*.] mutants. **(L)** Effect of SCPs on the lifespan of RB1985[*acox-1.5(ok2619) III*.] mutants. Different letters correspond to statistically significant differences (*p* < 0.05) between groups.

The GFP signal was shown as cytosolic, intermediate, and nuclear localization in Figure 6A. The results illustrated a significant rate of higher nuclear location up to 44% and lower cytosolic location down to 57% compared to the control (Figure 6B). Furthermore, SCPs had no significant effect on lifespan in *daf-16* null mutants, control (11.28 ± 0.34), SCPs-L (11.67 ± 0.35), SCPs-M (12.22 ± 0.06), SCPs-H (12.19 ± 0.21). These data suggested that the deletion of *daf-16* could affect the role of SCPs (Figure 6C). These results conveyed that SCPs might promote DAF-16 translocation to enhance the effects on stress resistance and longevity.

The inhibited *daf-2* or activated *daf-16* could be the key target for regulating IIS to delay aging in nematodes. SCPs could effectively reduce the accumulation of lipofuscin (Figures 6D,E) and extended lifespan in *daf-2* null mutants with no significant difference, control (22.78 ± 0.88), SCPs-L (20.72 ± 0.91), SCPs-M (22.80 ± 0.30), SCPs-H (22.11 ± 0.91), which suggested that *daf-2* played an important role in delaying aging in SCPs (Figure 6F). SOD-3 can remove excess superoxide and free radicals in the body (29). In comparison to the control group, SCPs-L, M, and H treatments increased the expression of SOD-3: GFP by 76.21, 90.98, and 26.89%, respectively (Figures 6G,H). SCPs had no significant effect on lifespan in *pept-1* null mutants, control (21.35 ± 0.83), SCPs-L (20.43 ± 0.88), SCPs-M (20.77 ± 0.26), SCPs-H (21.08 ± 0.16) (Figure 6I). OLD-1 was transcriptionally regulated by the DAF-16 forkhead transcription factor, and both were expressed in the whole body. SCPs were able to significantly reduce the lifespan of *old-1* null mutants compared with the control group, control (14.62 ± 0.55)^a^, SCPs-L (13.02 ± 0.23)^b^, SCPs-M (11.32 ± 0.12)^c^, SCPs-H (12.39 ± 0.25)^bc^ (Figure 6J). These results suggest that SCPs extend the *C. elegans* lifespan and improve antioxidant ability via *daf-16, daf-2, sod-3, pept-1*, and *old-1*.

SCPs up-regulated *tyr-3* and *acox-1.5* by other ways of anti-aging, which suggests SCPs might mediate the reduction of apoptosis and regulation of the Dauer phase in nematodes, by validating their corresponding mutants, we found that SCPs extended, respectively the lifespan of *tyr-3* null mutants 19.91, 14.72, 10.89% than the control group (Figure 6K). And also, SCPs extended the lifespan of *acox-1.5* null mutants, control (14.07 ± 0.21)^b^, SCPs-L (16.14 ± 0.20)^a^, SCPs-M (16.65 ± 0.11)^a^, SCPs-H (16.21 ± 0.13)^a^ (Figure 6L). This means SCPs are not able to act by inhibiting apoptosis and regulating the Dauer phase.

**Figure 7:**
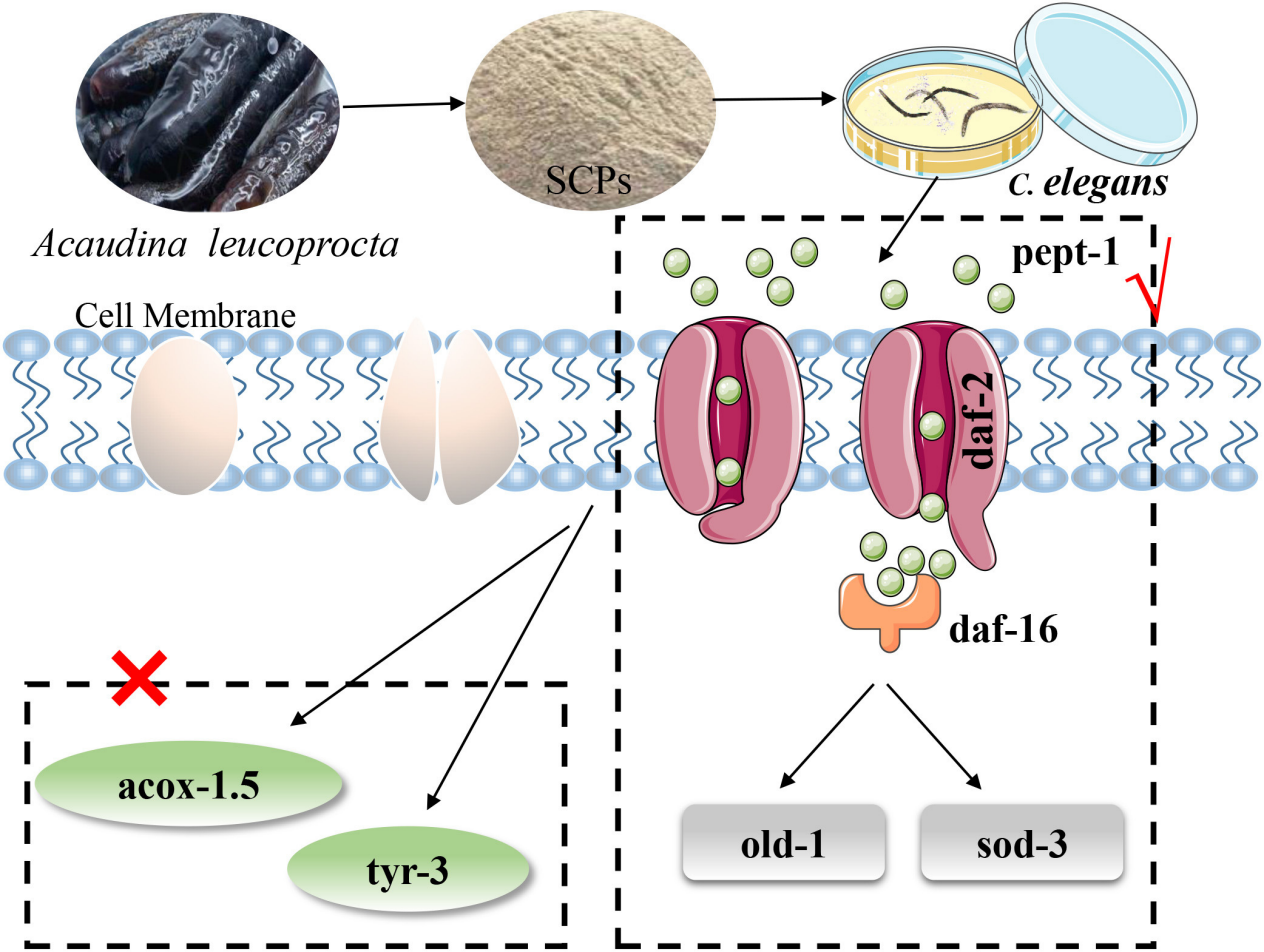
SCPs extended the lifespan of *C. elegans* could through the IIS pathway (*pept-1, daf-2, daf-16, old-1, sod-3*) but not apoptosis and Dauer phase.

In summary, the following diagram of the mechanism of SCPs regulating aging was obtained in Figure 7.

## Discussion

The population is aging, which is a severe issue in recent decades. Anti-aging product research is exploding, and biologically active peptides to slow down the aging process has become a hot topic. Bioactive peptides are effective and have been well studied, such as the rice bran peptide KF-8 improves the health span of *Caenorhabditis elegans* (30) and the walnut protein exhibited an excellent anti-photoaging effect (31). As examples of animal peptide sources, chicken bone collagen peptides have been shown to dramatically reduce the signs of skin aging (32), and the crimson snapper scales peptides effectively extended the lifespan and improved the motor ability of both male and female Drosophila (33). In the sea cucumber peptides research, SCPs (*Stichopus variegates*-derived peptides) significantly reduced D-gal-induced oxidative damage in mice by triggering SOD and GSH-Px and obstructing lipid peroxidation and protein oxidation (34, 35). Additionally, SCPs (*Apostichopus japonicas*-derived peptides) could also alleviate oxidative stress in neuroblastoma cells and improve survival exposed to neurotoxic paraquat in *C. elegans* (36). As a result, in the wide-type N2 and mutant model, we found that SCPs (*Acaudina leucoprocta*-derived peptides) have positive effects on health promotion, lifetime extension, and the likely underlying mechanism. As such, we detected the effects of SCPs on health promotion, lifespan extension, and the probable underlying mechanism in the wide-type N2 and mutant model. SCPs extend the lifespans of *C. elegans*, the mean lifespan of SCPs-M, and H were all higher than those of the positive Met, and the SCPs-M was higher than the NMN. The average lifespan of nematodes can be extended by up to 31.64% more than the 20% extension of sea cucumber *Apostichopus japonicus* (6). The longevity elevation of *C. elegans* is always accompanied by an improvement in stress resistance capacity (37). As anticipated, SCPs can significantly increase nematodes' resistance to heat and oxidative stimulation and decrease the build-up of lipofuscin and ROS in *C. elegans*. SCPs, like the sea cucumber *Apostichopus japonicus*, could boost SOD and CAT activities and decrease MDA accumulation in terms of anti-oxidation *in vivo*.

The IIS is characterized by diminishing insulin signaling, enhancing insulin sensitivity, and reducing plasma insulin-like growth factor-1 levels. This is a process involved in the formation of Dauer larvae (38). A DAF-16 transcription factor is crucial in regulating lifespan and stress resistance in nematodes (39). Under normal physiological activity, *daf-16* activity is at low levels, however, the *daf-2* mutation may restore *daf-16* activity, allowing adults to live more than twice as long as N2 (40). Therefore, we further studied the role of the insulin signaling pathway in regulating aging, and the results showed that treatment with SCPs improved the extension of the lifespan of *daf-2(e1370)* mutants, promoted the transfer of DAF-16 into the nucleus, up-regulated *pept-1* and increased the expression of the *daf-16* downstream gene, including *sod-3, old-1*. We have demonstrated that SCPs have exceptional resistance to oxidative stress. SCPs were able to increase the expression of the *sod-3* fluorescent protein and considerably reduce the lifespan of *old-1* null mutants as compared to the control group, but they had no effect on *pept-1* null mutants.

In addition, SCPs may delay aging through other pathways. According to research, TYR-2 and TYR-3 deficiency causes increased CEP-1 activation and germ cell death (41). Dauer pheromones or daumones, both of which were signal molecules that interrupted development and reproduction (dauer larvae) during unfavorable growth conditions in *C. elegans*. Acox-1 of nematodes was an essential component of daumone biosynthesis (42), and *acox-1.5* was an ortholog of human ACOX1 (acyl-CoA oxidase 1). In the current study, SCPs significantly up-regulated the expression of the *tyr-3, acox-1.5*, and extended the lifespan of the *tyr-3, acox-1.5* null mutants. Positive controls were used in the study, Met and NMN. Metformin regulated the insulin and IGF-1 signaling (43), which were similar effects that SCPs exerted on *C. elegans*. NMN is a widespread reduction in NAD^+^ that is connected to all of the signs and symptoms of aging. Age-related illnesses will be postponed or even reversed by restoring NAD^+^ levels (44). These results indicated that SCPs could extend the lifespan, improve the health span and enhance stress resistance possibly via the *daf-2*,*daf-16*,*sod-3*,*old-1*, and *pept-1* axis in *C. elegans*, and to our knowledge, biologically active peptides to be studied in depth on *pept-1* expression, *old-1* is a gene that effectively regulates aging. This study is a new direction for the research of bioactive peptides on the development of peptides from sea cucumber (*Acaudina leucoprocta*) and the development and application of dietary sea cucumber peptides in mechanism research. The insulin pathway was given a new direction in particular. However, the resource utilization of *Acaudina leucoprocta* still needs to be enhanced and research ideas need to be further developed.

## Conclusion

In this study, we have shown that SCPs from *Acaudina leucoprocta* increased lifespan and motility. SCPs not only increase the resistance of *C. elegans* to stress (heat and oxidative stress) but also inhibit lipofuscin and ROS accumulation, upregulate SOD and CAT activities, and reduces MDA content in *C. elegans*. Nematodes pretreated with SCPs showed increased expression levels of anti-aging genes (*daf-16, sod-3, pept-1, old-1, tyr-3, acox-1.5*), and decreased expression of *daf-2*. More than that, SCPs promote the migration of DAF-16 into the nucleus. SCPs extend the lifespan of *daf-2 (e1370)*, and activate *sod-3* green fluorescent protein expression and SCPs had no significant effect on lifespan in *pept-1* null mutants and were able to significantly reduce the lifespan of *old-1* null mutants. SCPs prolong the lifespan of *tyr-3* and *acox-1.5* null mutants. In summary, these results suggest that SCPs could extend the lifespan and enhance antioxidant capacity via DAF-16/DAF-2/SOD-3/OLD-1/PEPT-1 not TYR-3/ACOX-1.5 in *Caenorhabditis elegans*.

## Data availability statement

The original contributions presented in the study are included in the article/Supplementary material, further inquiries can be directed to the corresponding authors.

## Author contributions

YW, JY, and CX designed experiments, carried out the experiments, analyzed the experimental results, prepared the original draft, and performed the statistical analysis. QL, YM, and SZ finished the validation. YW, JZ, FS, QW, and XZ reviewed and edited the manuscript. XZ and FF acquired resources. All authors contributed to the article and approved the submitted version.

## Funding

Major Project of Science and Technology Department of Yunnan Province (202002AA1000055). The Yunnan Young and Elite Talents Project (YNWR-QNBJ-2020-213).

## Conflict of interest

The authors declare that the research was conducted in the absence of any commercial or financial relationships that could be construed as a potential conflict of interest.

## Publisher's note

All claims expressed in this article are solely those of the authors and do not necessarily represent those of their affiliated organizations, or those of the publisher, the editors and the reviewers. Any product that may be evaluated in this article, or claim that may be made by its manufacturer, is not guaranteed or endorsed by the publisher.
