# Supplementary material for: Sea cucumber (Acaudina leucoprocta) peptides extended the lifespan and enhanced antioxidant capacity via DAF-16/DAF-2/SOD-3/OLD-1/PEPT-1 in Caenorhabditis elegans
Source: Front Nutr. 2022 Nov 22;9:1065145. doi: 10.3389/fnut.2022.1065145 (PMC9723373; doi:10.3389/fnut.2022.1065145)
Supplement: Supplementary file 1 [file Presentation_1.pptx]

## Slide 1
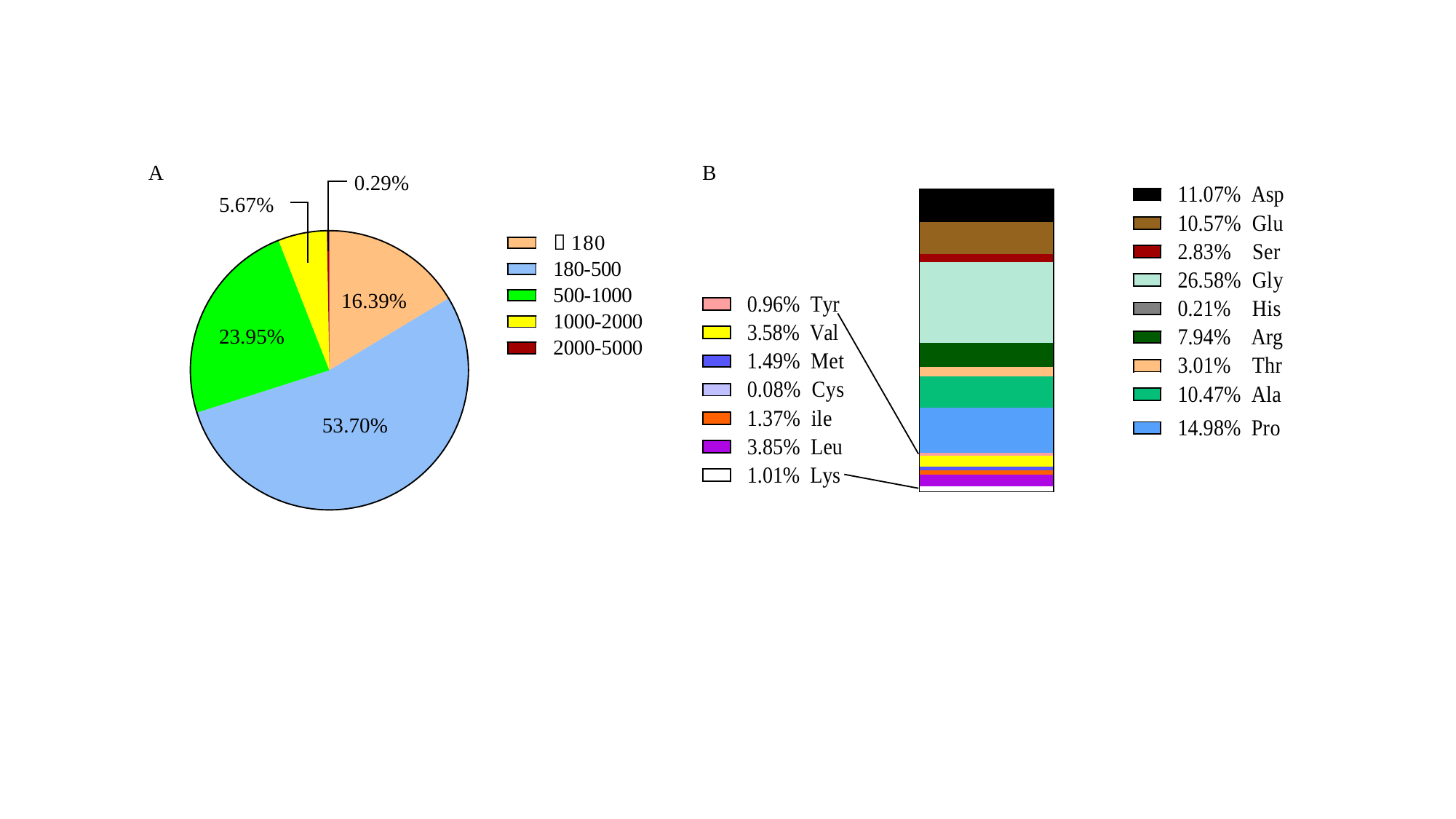

A
B

## Slide 2
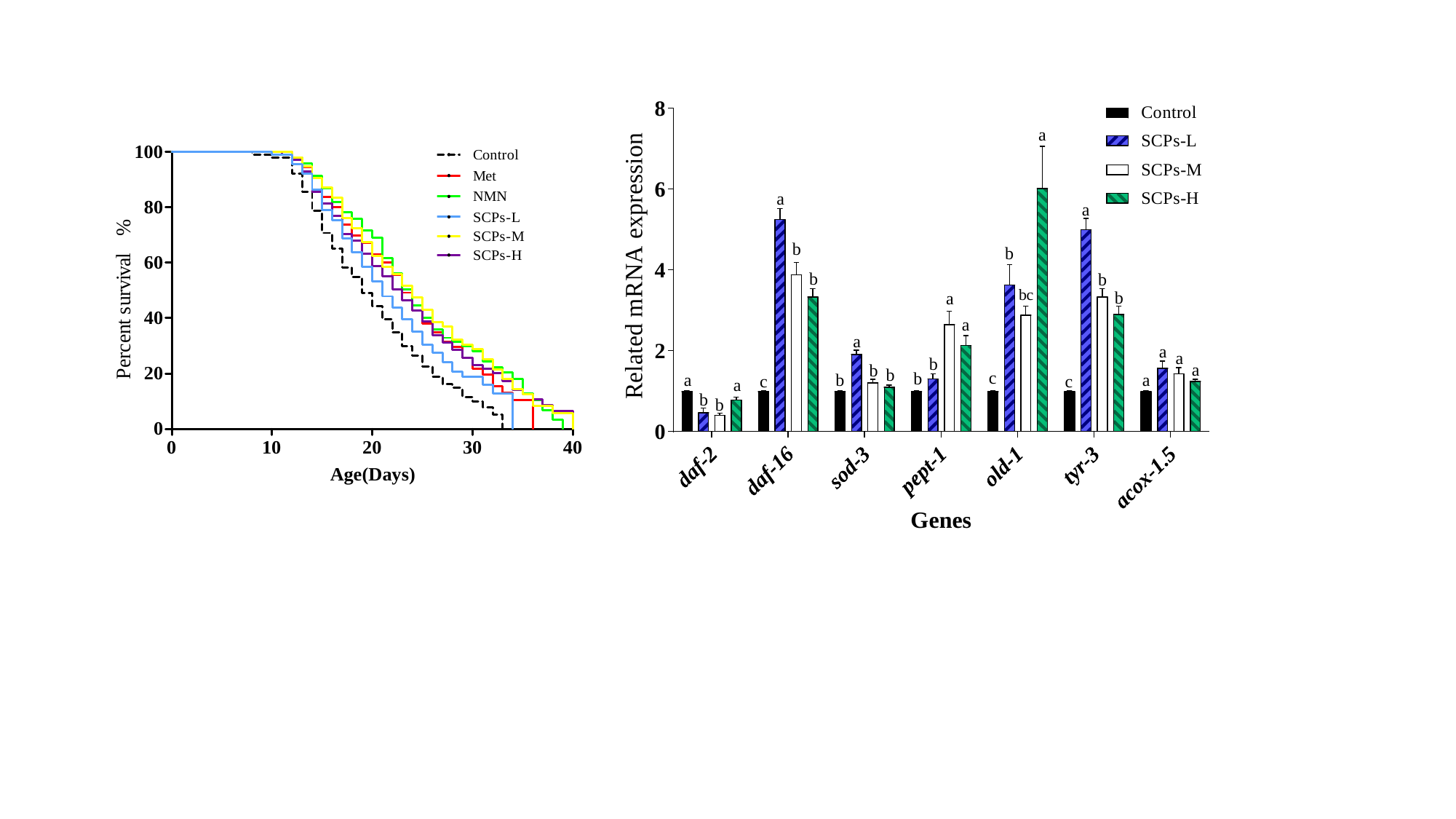

## Slide 3
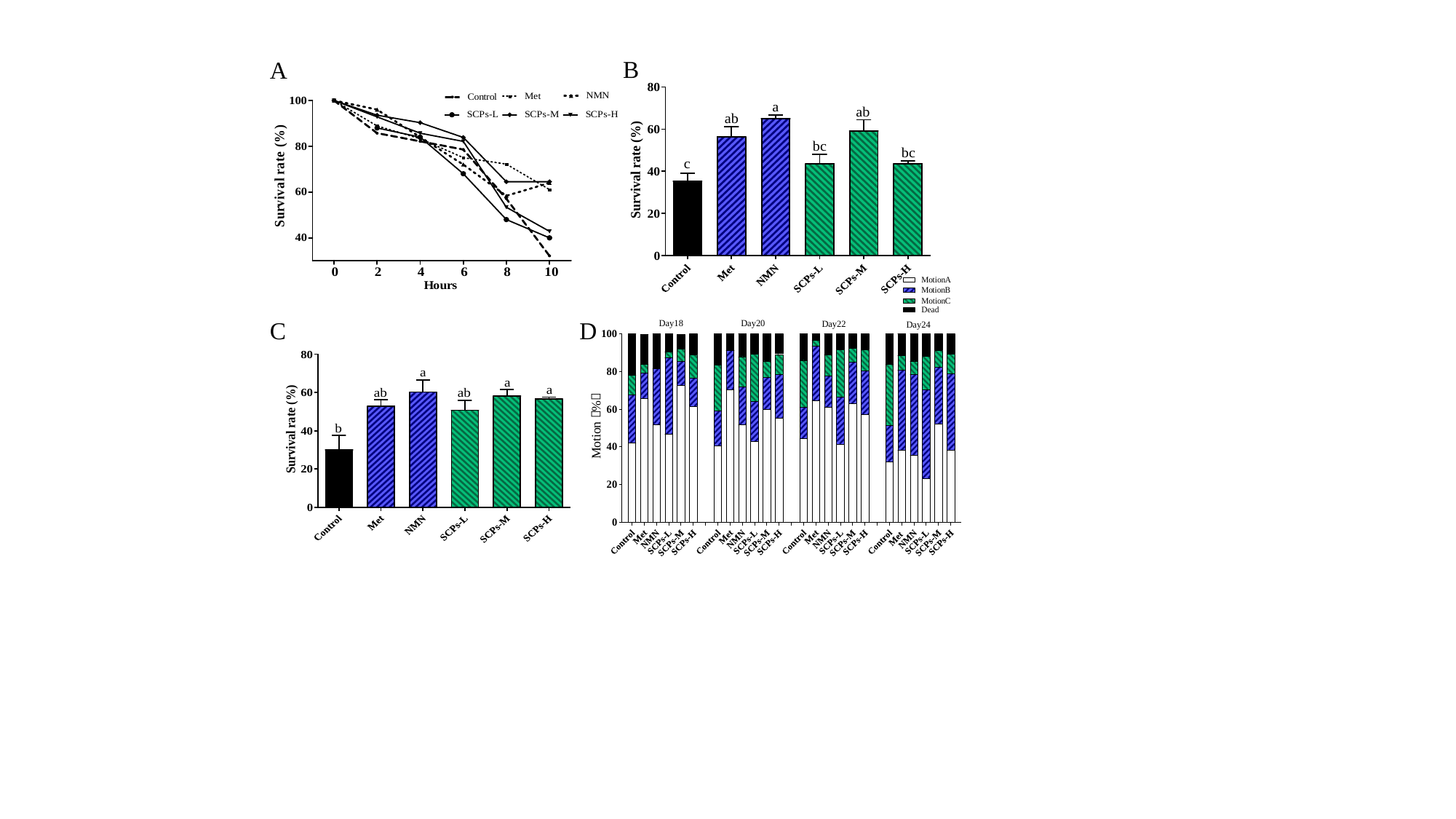

A
C
D
B

## Slide 4
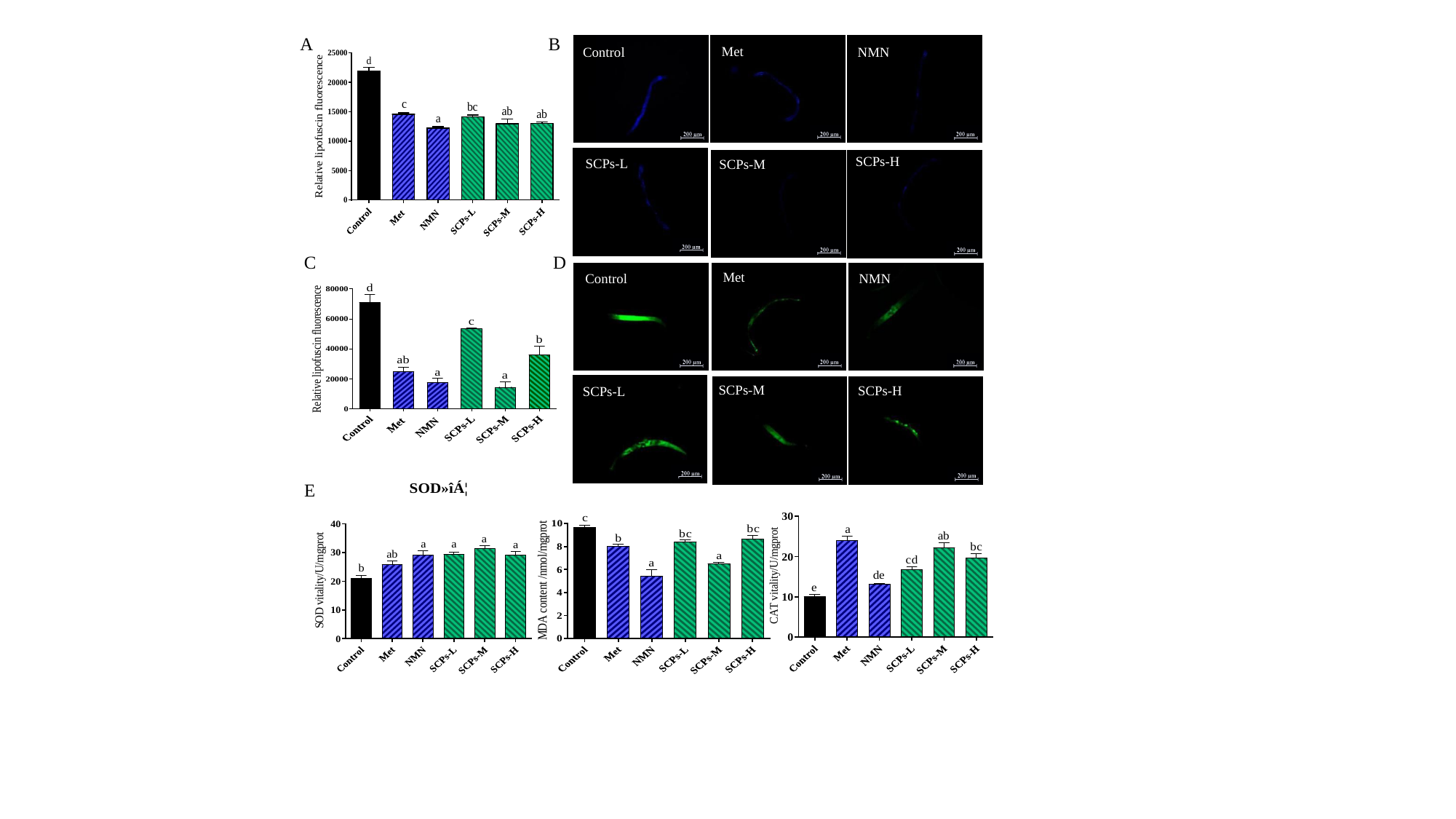

B
A
Met
Control
NMN
SCPs-H
SCPs-L
SCPs-M
D
C
Met
NMN
Control
SCPs-M
SCPs-H
SCPs-L
E

## Slide 5
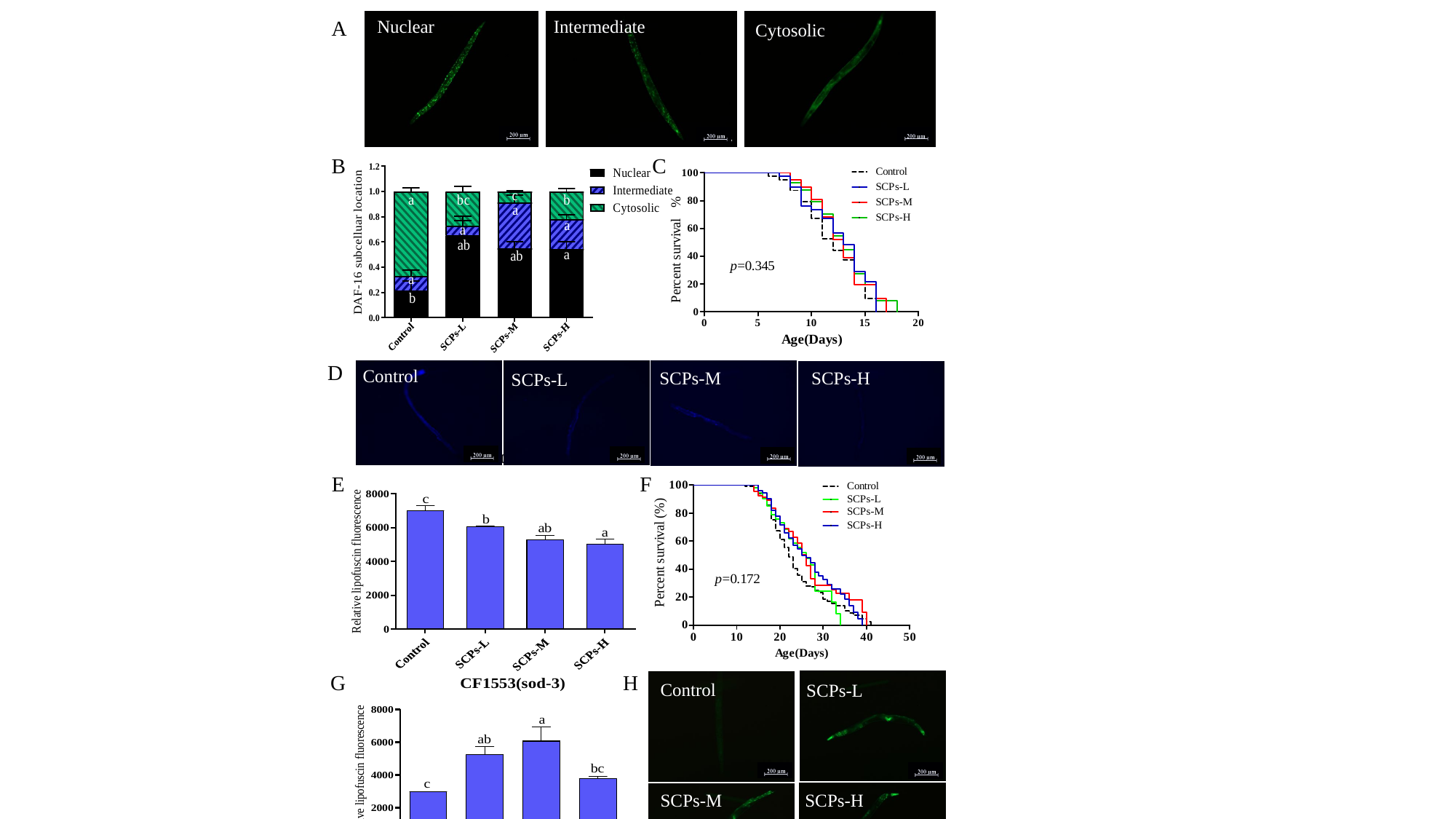

A
Intermediate
Nuclear
Cytosolic
B
C
D
Control
SCPs-L
SCPs-M
SCPs-H
E
F
G
H
SCPs-L
Control
SCPs-H
SCPs-M

## Slide 6
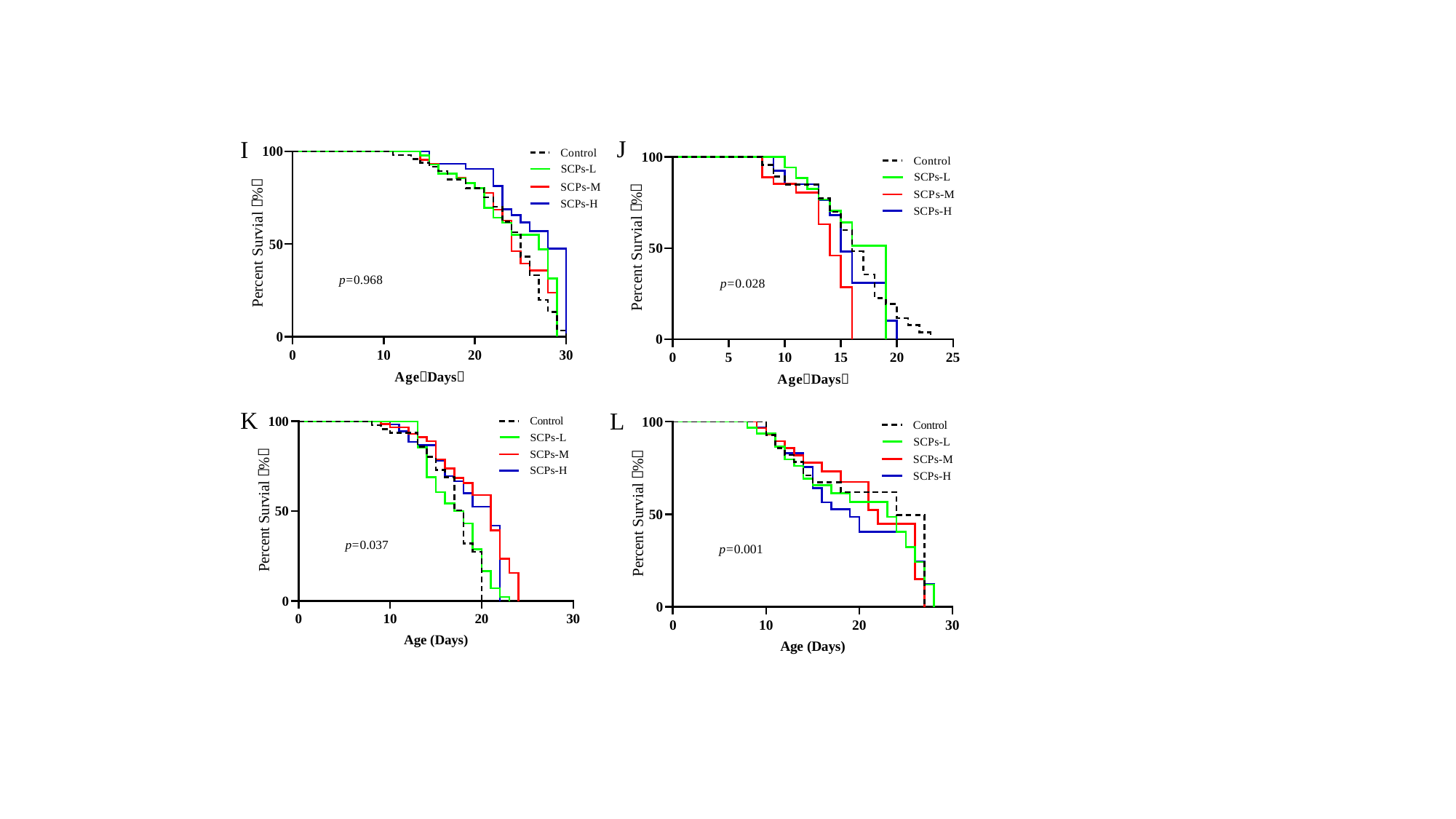

J
I
K
L

## Slide 7
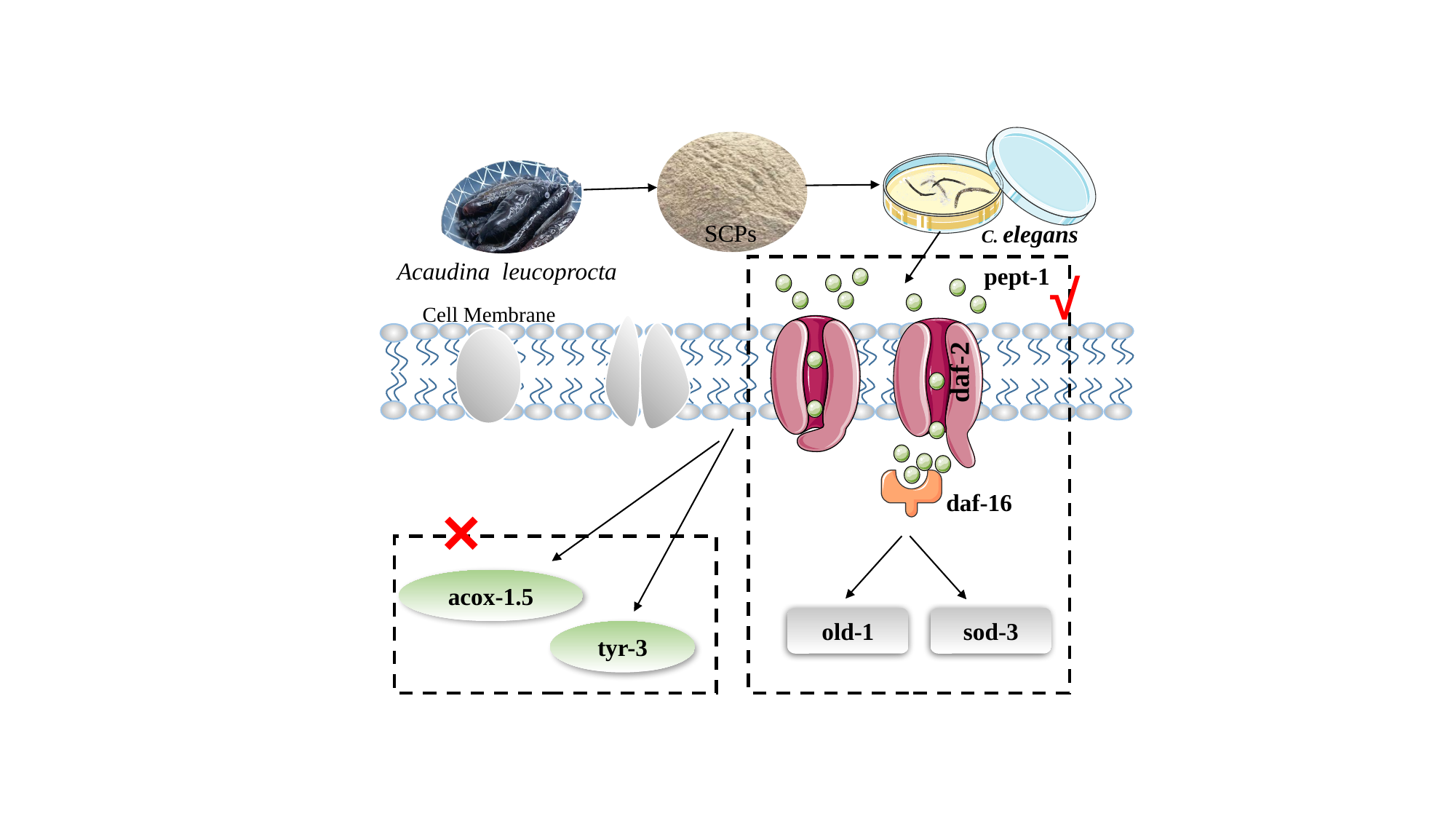

SCPs
C. elegans
Acaudina leucoprocta
pept-1
√
Cell Membrane
daf-2
daf-16
×
acox-1.5
old-1
sod-3
tyr-3
